# An Ecosystem Evaluation Framework for Global Seamount Conservation and Management

**DOI:** 10.1371/journal.pone.0042950

**Published:** 2012-08-08

**Authors:** Gerald H. Taranto, Kristina Ø. Kvile, Tony J. Pitcher, Telmo Morato

**Affiliations:** 1 Departamento de Oceanografia e Pescas, IMAR, LARSYS, Universidade dos Açores, Horta, Portugal; 2 Fisheries Center, University of British Columbia, Vancouver, Canada; Aristotle University of Thessaloniki, Greece

## Abstract

In the last twenty years, several global targets for protection of marine biodiversity have been adopted but have failed. The Convention on Biological Diversity (CBD) aims at preserving 10% of all the marine biomes by 2020. For achieving this goal, ecologically or biologically significant areas (EBSA) have to be identified in all biogeographic regions. However, the methodologies for identifying the best suitable areas are still to be agreed. Here, we propose a framework for applying the CBD criteria to locate potential ecologically or biologically significant seamount areas based on the best information currently available. The framework combines the likelihood of a seamount constituting an EBSA and its level of human impact and can be used at global, regional and local scales. This methodology allows the classification of individual seamounts into four major portfolio conservation categories which can help optimize management efforts toward the protection of the most suitable areas. The framework was tested against 1000 dummy seamounts and satisfactorily assigned seamounts to proper EBSA and threats categories. Additionally, the framework was applied to eight case study seamounts that were included in three out of four portfolio categories: areas highly likely to be identified as EBSA with high degree of threat; areas highly likely to be EBSA with low degree of threat; and areas with a low likelihood of being EBSA with high degree of threat. This framework will allow managers to identify seamount EBSAs and to prioritize their policies in terms of protecting undisturbed areas, disturbed areas for recovery of habitats and species, or both based on their management objectives. It also identifies seamount EBSAs and threats considering different ecological groups in both pelagic and benthic communities. Therefore, this framework may represent an important tool to mitigate seamount biodiversity loss and to achieve the 2020 CBD goals.

## Introduction

Deep-sea and open ocean waters are the largest and yet least understood environments on Earth [1], [2]. They are characterized by distinctive habitats and organisms and support an important part of the world’s biodiversity [1], [3]. Moreover, these ecosystems provide valuable direct and indirect goods and services, such as food provision and climate regulation [4]. In the last decades, the human pressure on these systems has sharply increased [5] threatening their health, biodiversity and resilience. In fact, the decrease of natural and mineral resources on land and in shallow waters, coupled with a rapid technological development which now allows the exploitation of formerly inaccessible areas, has caused a constant expansion of human-related activities toward deeper and more distant areas [6], [7]. Therefore, appropriated forms of governance and management of deep and open ocean ecosystems are essential to preserve their structures, processes and the services they provide.

One of the major challenges in designing and implementing governance and management strategies in these environments is the fact that deep seas and open oceans frequently fall in areas beyond national jurisdiction (ABNJ). Therefore, international commitments are necessary to undertake effective conservation actions. In the last twenty years, several global meetings on biological conservation and sustainable development have proposed to set aside for protection 10–30% of all the marine biomes (including deep sea and open ocean realms) by the year 2012. The failure in meeting these objectives has been internationally recognized with just 1.17% of the world’s oceans currently included in marine protected areas (MPAs) mostly located in coastal waters [8]. Revised biodiversity targets aiming at preserving 10% of all the marine biomes by 2020 were agreed at the 10th Convention of the Parties (COP) to the Convention on Biological Diversity (CBD). In order to speed up deep sea and open ocean conservation and achieve the proposed targets, the Parties to the CBD have adopted in 2008 seven scientific criteria for identifying ecologically or biologically significant areas (EBSA) in need of protection in open-ocean waters and deep-sea habitats (COP decision IX/20 paragraph 14). These criteria are: uniqueness or rarity; special importance for life-history stages of species; importance for threatened, endangered or declining species and/or habitats; vulnerability, fragility, sensitivity or slow recovery; biological productivity; biological diversity; and naturalness (Table 1) [9]. The application of the CBD EBSA criteria should ultimately allow the establishment of representative marine protected area networks in the high seas and help the implementation of ecosystem based managements. These networks should cover a full range of examples across biogeographic regions as defined, for example, in the Global Open Ocean and Deep Sea (GOODS) lower bathyal biogeographic classification [10]. CBD also defined five criteria for the definition of representative networks of MPAs: identification of ecologically or biologically significant areas, representivity, connectivity, selection of replicated ecological features and selection of viable and adequate sites [9]. The identification of ecologically and biologically significant seamount areas and the selection of the seamounts more suitable for conservation may represent an important first step in the creation of such networks. However, the present framework is intended to be applied to individual seamount features and not for the identification of networks of such sites.

**Table 1:** Application of the scientific criteria adopted for identifying ecologically or biologically significant areas to seamounts ecosystems.

EBSA Criteria	Description	Seamount EBSA Indicator	Ref.
Uniqueness or rarity	Area contains either (i) unique, rare or endemic species,populations or communities, and/or (ii) unique, rare or distinct, habitats or ecosystems; and/or(iii) unique or unusual geomorphological or oceanographic features	Vents communities, macrophytes, datarich supportedcases of endemism	[62], [63], [106], [105]
Special importance for life-history stages of species	Areas that are required for a population to survive and thrive	Aggregating deep-sea fishes, air-breathing visitors, large visiting pelagics	[18], [19], [31]–[34], [47], [71]–[74], [107]
Importance for threatened, endangered or declining species and/or habitats	Area containing habitat for thesurvival and recovery of endangered, threatened, decliningspecies or area with significant assemblages of such species	Habitat-formingcold water corals, sponge aggregations, threatened bottomfish and sharks, threatened air-breathing visitors, threatened visiting pelagics	[50], [75], [76]
Vulnerability, fragility, sensitivity, or slow recovery	Areas that contain a relatively high proportion ofsensitive habitats, biotopes or species that are functionally fragile(highly susceptible to degradation or depletion byhuman activity or by natural events) or with slow recovery	Habitat-formingcold water corals, sponge aggregations, vents communities,aggregating deep-sea fishes	[44]–[47], [50], [53], [78], [86], [108]
Biological productivity	Area containing species, populations or communities with comparatively higher natural biological productivity	Macrophytes,vents communities,“shallow” seamounts	[29], [62], [63], [81]–[83], [109]
Biological diversity	Area contains comparativelyhigher diversity of ecosystems, habitats, communities,or species, or has higher genetic diversity	Habitat-formingcold water corals, macrophytes,sponge aggregations	[77], [84]–[86], [109], [110]–[115]
Naturalness	Area with a comparativelyhigher degree of naturalness asa result of the lack of or lowlevel of human-induced disturbance or degradation	No fishing or mining impact	

The indicators chosen to assess each criterion for seamount ecosystems and references supporting their choice are shown.

The methodologies suggested by the CBD should not be restricted to ABNJ and could also be adopted and implemented within areas of national jurisdiction. Pilot studies have identified several potential EBSAs in different marine regions (www.gobi.org). However, the patchy nature of biological and ecological data regarding deep and open ocean ecosystems hinders a systematic application of these criteria and implies a wide reliance on global models and remote sensed data [11]. Moreover, areas of critical importance in the water column tend to shift in time and space, making the location of pelagic EBSAs even more difficult [12].

Dynamic marine protected areas have been suggested as tools for conserving open ocean biodiversity [13]–[14]. However, there has been some debate on their workability and utility questioning the possibility of a rapid implementation of pelagic MPAs in real world conservation actions [15]–[17]. Thus, non-dynamic features such seamounts and ridges may represent good starting points for a systematic implementation of offshore marine reserves, since they have been demonstrated to be easier to conserve, map, survey, and enforce than ephemeral areas. At the same time conservation of seamount ecosystems seems to be beneficial both for benthic and pelagic organisms [18], [19].

Seamounts are prominent and ubiquitous features of the world’s underwater topography [20], [21] and constitute one of the largest biomes of the deep-sea [22]. Several authors have illustrated their importance for the benthic and pelagic realms. For example, Samadi et al. [23] found an increased species richness and abundance of galatheid crabs on seamounts and proposed that benthic invertebrates are more abundant and attain higher diversity on submarine reliefs compared to other deep-sea habitats (‘oasis hypothesis’). The high densities of filter feeders, especially corals and sponges, that can be encountered on seamounts seem to confirm the oasis hypothesis [24]–[26], even though no robust quantitative estimates are currently available [27], [28]. The interaction of seamounts with vertically migrating organisms and passing oceanic flows appears to facilitate trophic exchanges toward top pelagic predators [29]. Therefore, seamounts seem to be important hotspots for pelagic biodiversity and visitor organisms and play an important role in enhancing fishery catches of some pelagic species [18], [19], [30]–[37]. In Morato et al. [19], in particular, the aggregating behavior of large pelagic fish, both visitors and not, was showed to be diffuse throughout Southwest Pacific seamounts and to occur within 30–40 km of seamount summits. However, seamounts are very heterogeneous habitats and the above mentioned properties may not be common to all submarine features [38]. In fact, seamounts are generally characterized by diverse geophysical properties, which in turn are likely to affect the biological diversity and production of resident and associated organisms [29], [30], [38]–[40]. As a consequence, the protection of different seamounts may ultimately result in very different outcomes. The use of the CBD EBSA criteria can help to identify seamounts more likely to be suitable for protection.

Although the CBD EBSA criteria suite represents a powerful tool in identifying areas of particular ecological or biological importance, parallel socio-economic and governance analysis are needed if marine policies are to find a balance among multiple ecological, socio-economic and other governance objectives [41]. An important part of this process is represented by a correct definition and measurement of the major human activities occurring in these areas. Fishing is considered one of the major threats to seamount ecosystems [42], [43] having long-term impacts on different habitats, such as coral and sponge aggregations e.g. [44]–[46] and on vulnerable, long-lived fish stocks e.g. [47]–[49]. The detrimental effect of fishing on seamounts has been stressed in the FAO guidelines for sustainable fishing [50], where submarine elevations are listed as an example of vulnerable marine ecosystems. Besides fisheries, deep-sea mining is emerging as an important issue in seamount management [51]. Different types of metal-rich deposits can be found on seamounts, of which Fe-Mn crusts and massive polymetallic sulphide are of highest commercial interest [52]. Even though no substantive exploitation has started, with the exception of few exploratory surveys, mining activities on submarine features are likely to pose a serious threat to seamount ecosystems in the near future [53], [54].

In this study we propose a framework for applying the CBD EBSA criteria to locate potential ecologically or biologically significant seamount areas, based on the best information currently available. In particular, this work developed methods for applying the EBSA criteria to individual seamounts and methods to assess the impact of different fishing gears and mining activities on the various components of individual seamounts such as pelagic, benthopelagic and benthic environments. This framework will allow managers to identify EBSAs and to prioritize their choices or policies in terms of protecting undisturbed areas, protecting disturbed areas for recovery of habitats and species, or both. CDB prioritize areas having low levels of disturbance relative to their surroundings. However, where no natural areas remain, areas with high possibilities of recovery after the cease of anthropogenic related activities should be considered [9], [55]. Thus, measuring major human activities is of paramount importance in the seamount conservation process. The application of the present framework to seamounts and the possibility to redesign it for other habitats (e.g., hydrothermal vents, pelagic fronts, etc.) could strongly enhance a systematic approach to deep sea and open ocean management. The outcomes of these evaluations should serve as a powerful tool for identifying sites of particular importance for conservation which can then be integrated in MPA networks following the set of principles and criteria guiding design and implementation of MPA networks e.g. [9], [41], [56].

## Methods

The framework proposed in this study for assessing seamount EBSAs was developed within the conservation part of the Seamount Ecosystem Evaluation Framework, SEEF [57]–[59] and consists of both a semi-quantitative scoring of the biological value individual seamounts have with respect to the EBSA criteria and an evaluation of the main threats posed to each seamount. The overall EBSA and threats scores can then be used to visualize all seamounts on a scale ranging from low to high likelihood of being an EBSA and low to high threats allowing for the comparison of different features. Different definitions of the term seamount are available in literature [60]: here, seamounts are considered as topographically distinct seafloor features greater than 100 m in height but which do not break the sea surface [61].

### Application of the EBSA Criteria to Seamount Ecosystems

The relevance of individual seamounts with respect to the different EBSA criteria were assessed based on the presence of particular habitats, communities and species (i.e. indicators) that capture the most relevant pelagic, benthopelagic and benthic components of seamount ecosystems. The presence of such indicators can be assessed by both using real data coming from sampled features or global models which could complement data deficient sites.

#### Uniqueness or rarity (C1, Table 1)

The presence of hydrothermal vents on seamounts was used as a proxy for uniqueness. In fact, vents host a number of special communities and organisms that are found nowhere else in the marine environment e.g. [62], [63]. A second factor used to assess seamounts uniqueness was the presence of macrophytes. Benthic primary producers are extremely rare in the open oceans and therefore represent a valid proxy for rarity. This indicator has already been proposed to candidate the Saya de Malha Banks as an EBSA (http://www.gobi.org/candidate-ebsas). The presence of endemic organisms to assess the level of faunal uniqueness has to be considered with extremely caution, since seamount endemicity has been recently questioned [23], [64]–[67]. In fact, the exploration of new seamount areas is generally followed by the description of several endemic species e.g. [68], [69] which tend to keep their endemic status only until new studies with wider and more detailed spatial and taxonomic coverage are performed e.g. [23]. The number of endemisms may thus be directly related to sampling effort [70]. Therefore, considering the difficulty in discriminating between true and apparent endemism, it was decided to consider this indicator in the uniqueness or rarity criteria but its implementation will not be done until more clues on the seamount endemicity hypotheses are revealed.

#### Special importance for life-history stages of species (C2, Table 1)

Areas containing breeding or spawning grounds, juvenile habitat and important habitats for migratory species are considered good examples of this criterion [9]. Seamounts represent an important feeding and/or spawning ground for a number of different seamount-associated fishes. These species are known in literature as “aggregating deep sea fishes” and are described and listed in Koslow [71] and Morato et al. [47]. Furthermore, seamounts play an important role for large visiting pelagic species (i.e., tunas, billfishes and large pelagic sharks) e.g. [18], [19], [35], [72], [73] and air-breathing visitors (i.e., marine mammals, marine turtles and seabirds) e.g. [18], [33], [34], [74]. Therefore, the presence of aggregating deep sea fishes, large visiting pelagics and air-breathing visitors was used to identify areas with special importance for life-history stages of species.

#### Importance for threatened, endangered or declining species and/or habitats (C3, Table 1)

The IUCN red list provides a comprehensive list of threatened, endangered or declining species [75]. Air-breathing visitors, large visiting pelagic and bottom fish and shark species which are included in the red list as critically endangered, endangered, vulnerable or nearly threatened were used to assess this criterion. Thus, when such species are reported for a seamount, this location is considered important for threatened species or habitats. In addition, the presence of habitat-forming cold water corals and sponge aggregation which represent declining habitats e.g. [76] was also reckoned to be relevant to this criterion and included in the present analysis. It is important to notice that the cold water corals used here as a proxy for criteria 3, 4 and 6 are exclusively those forming deep-water reefs and gardens. A list of structure-forming corals was adapted from Roberts et al. [77] and is provided as Supporting Information (Table S1).

#### Vulnerability, fragility, sensitivity, or slow recovery (C4, Table 1)

This criterion values the degree of risk that will be incurred from human activities or natural events [9]. Coral gardens and reefs, sponge aggregations and vent communities are highly sensible to human disturbance and are listed in international guidelines as examples of vulnerable marine ecosystems deserving particular protection [50], [76]. Besides these groups, aggregating deep-sea fishes *sensu* [71] were considered in the assessment of this criterion since these species are long-lived and slow-reproducing organisms extremely vulnerable to human disturbance [47], [49], [78].

#### Biological productivity (C5, Table 1)

The dynamics of marine production in the open and deep oceans are poorly understood. To date, the depth of submarine features represents the best indicator of seamount productivity. In fact, shallow seamounts may intercept the diel vertical migration of zooplankton and micronekton, trapping these vertically migrating organisms and/or aggregate small zooplanktonic animals horizontally advected. This could result in an increased prey availability, which may benefit resident and visiting animals enhancing secondary production and aggregating behaviors [28], [29], [79], [80]. The lower range of these migrations is thought to range between 400 and 800 m [29], [81]. Therefore, seamounts shallower than 800 m were assumed to have a higher productivity than deeper features. In addition, metazoan meiofauna, macrofauna and megafauna abundance and biomass tend to decrease sharply with depth as a consequence of limited nutrient availability in deeper water [82]. Therefore, deeper seamounts are likely to be less productive than shallower ones. Benthic primary producers (macrophytes) and hydrothermal vent communities were also regarded as indices of high biological production e.g. [63], [83].

#### Biological diversity (C6, Table 1)

Reliable estimates of biodiversity for seamounts are difficult to obtain with the data currently available in the scientific literature. However, the presence of structural species may increase local biological diversity and be used as a proxy for this criterion e.g. [84]–[86]. Therefore, the presence of seamount habitats dominated by cold water corals, sponges or macrophytes was used to assess this criterion.

#### Naturalness (C7, Table 1)

These are areas with a comparatively higher degree of naturalness as a result of the lack or low level of human disturbance [9]. The naturalness of individual seamounts depends on the typology and intensity of the anthropogenic activities over time. In the present work we considered a seamount to have a high degree of naturalness when no fishing or mining activities were known to occur. It is well recognized that different fishing activities have very different impacts in the ecosystem [87]. This distinction was taken in consideration when quantifying the human-induced disturbances on seamount ecosystems.

### Seamount EBSA Scoring Procedure

Ten indicators were used to identify seamount EBSAs (Table 2): four benthic (hydrothermal vents, macrophytes meadows, cold water corals, sponge aggregations), two benthopelagic (aggregating deep-sea fishes and threatened bottom fish/sharks), two pelagic (large visiting pelagic and air-breathing visitors), one historical (naturalness) and one geological (depth). The different proportion of indicators adopted for each seamount component reflects the relative importance benthic, benthopelagic, pelagic, geological and historical indicators had in the individuation of potential seamount EBSAs.

**Table 2:** Typology and weight of the indicators used to identify seamount ecologically or biologically significant areas.

Typology	EBSSA Indicator	Weight
Benthic	1) Hydrothermal vents	3
	2) Macrophytes	3
	3) Cold water corals	3
	4) Sponge aggregations	3
Benthopelagic	5) Aggregating deep-sea fishes	2
	6) Threatened bottom sharks and fishes	1
Pelagic	7) Threatened air-breathing visitors **OR** air-breathing visitors	2 **OR** 1
	8) Threatened visiting large pelagics **OR** visiting large pelagics	2 **OR** 1
Historical	9) Naturalness	1
Geological	10) Depth	1

Individual indicators were weighted based on the relevance they have in the assessment of the EBSA criteria. Factors used to verify more than one criterion had a higher weight in the analysis (Table 2). For example, the presence of habitat-forming cold water corals was used as a proxy for criteria 3, 4 and 6 and thus was weighted three times higher than depth, which was considered only for criterion 5. Visiting pelagic and air-breathing visitors were generally used as a proxy for criterion 2. However, if the large pelagic or air-breathing species present were listed in the IUCN red list as critically endangered, endangered, vulnerable or nearly threatened [75] they became relevant both for criterion 2 and 3 and their weight was doubled in the scoring process. The final score represents an index of the likelihood of having ecologically or biologically significant seamount areas on a particular seamount and was calculated based on the proportion of indicators present and on their weight in the analysis. It can range from 1 when no indicator is present to 5 when all indicators are present at a specific seamount. The final outcomes were presented as two nominal categories, from here on referred as “seamount EBSA likelihood score”, indicating the chance of having EBSAs on the assessed seamounts. These categories were: low, for total scores ≤3 and high, for total scores >3.

### Quantify Human-induced Threats to Seamount Ecosystems

The major human activity currently impacting seamounts is fishing [42], [49] and its effects are highly dependent on the type of fishing gear used [88], [89]. The main fisheries occurring on seamounts use trawls, longlines, gillnets and pots and traps [90] but other methods such as hook and line are also present in some small-scale seamount fisheries [91]. In addition to the type of fishing gear used, fishing effort (duration and frequency of fishing events) and catch data (landings, bycatch and discards) are essential to determine the actual impact of any fishery [92]. However, considering the lack of specific data regarding seamount fisheries, it is generally not possible to consider catch data and fishing effort. Therefore, the evaluation of fishing impacts on individual features was here exclusively based on the types of fisheries present.

Mineral exploitation is likely to pose a serious threat to seamount ecosystems in the near future [53], [54] and was therefore included in the evaluation as a potential threat factor. Finally, climate change will probably constitute the greatest threat to aquatic ecosystems [7]. Meanwhile, considering our poor understanding of the repercussions it will have on deep-sea organisms and habitats and the consequent difficulties in quantifying the effects of phenomena such as ocean acidification and hypoxia, it was decided not to implement climate change in the present analysis. A revision of the present framework should be considered as soon as new studies will clarify the consequences climate change will have on seamount ecosystems.

### Scoring Procedure for Human-induced Threats to Individual Seamounts

**Figure 1:**
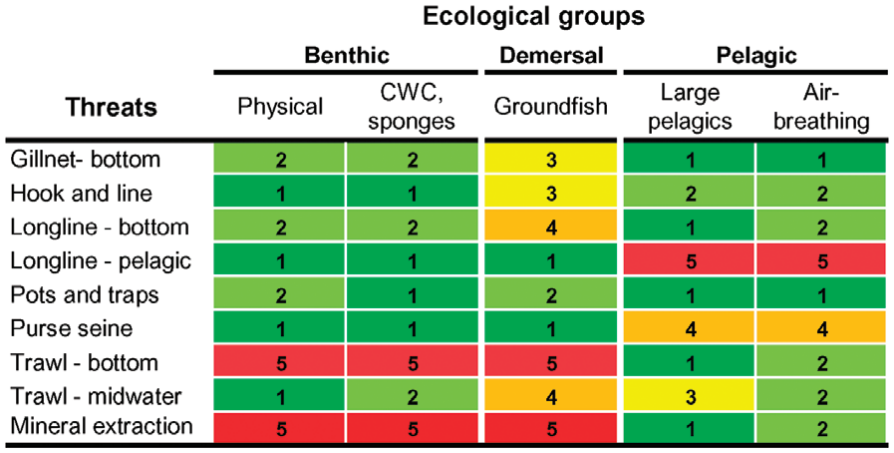
Impact of some anthropogenic activities on seamount ecosystems. A scoring system for the impact of fishing gears and mining activities on five ecological groups are shown (adapted from [81], [87]). The impacts are defined as: 1, very low; 2, low; 3, medium; 4, high; and 5, very high.

The effects of fishing and mining on individual seamounts were quantified using an expert knowledge system. This system was adapted from two recent reviews where experts were asked to rate the impact of several fishing gears on different taxonomic groups and habitats using a scale ranging from very low to very high [87], [93]. The set of ratings, threats and ecological groups considered in these studies were revised in order to obtain categories and scores more meaningful for seamount ecosystems. A total of nine types of threats (1 mining and 8 fishing activities) were regarded as particularly relevant for seamounts and included in the framework: bottom gillnet, hook and line, bottom and pelagic longline, pots and traps, purse seine, midwater and bottom trawl, seafloor mineral extraction. Five potentially threatened components of seamount ecosystems were identified: two benthic (physical habitat and habitat-forming corals and sponges), one benthopelagic (groundfish) and two pelagic (large pelagic fish and air-breathing visitors, i.e. marine mammals, marine turtles and seabirds). The impact of each fishing gear and mining activity on each ecological group was rated on a scale from 1 -very low- to 5 -very high- (Figure 1). These ratings ultimately resulted in a weighting system for each human activity where, for example, bottom trawling poses a different degree of threat to seamount ecosystems than pelagic longlining.

The “threats score” of individual seamounts was determined by the anthropogenic activities occurring on that features. In fact, benthic, benthopelagic and pelagic seamount components will experience different levels of disturbance depending on the set of human related activities present. Among the set of fishing and mining practices present on a specific feature, it is possible to identify a subset of activities likely to pose the highest impacts to the different components considered (i.e. the ones posing the greatest threat to physical habitat, the ones posing the greatest threat to habitat-forming corals and sponges, etc). The final threat score was thus calculated as the average of the maximum impacts (Figure 1) posed to the different ecological groups and therefore it takes into account the benthic, benthopelagic and pelagic statuses of all the evaluated seamounts (see Information S1). The threats score range from 1 when no activity is present to 5 when the activities present potentially pose very high impacts on all the considered ecological groups. The final “threats score” (TS) were presented as three nominal categories: 1) none (TS = 1), seamounts with no anthropogenic impacts; 2) low (1<TS≤3), anthropogenic activities do not have severe impacts on any components of the seamount ecosystem, have moderate impacts on the seamount ecosystem, or impact severely only one component; 3) high (TS>3), anthropogenic activities are impacting several components of the seamount ecosystem and more than one is severely affected or have severe impacts on all the considered components of the seamount ecosystem.

### Data Uncertainty Index

To account for data uncertainty, data quality issues and the varying degree of knowledge regarding different seamounts and geographical area, a data uncertainty index similar to the one elaborated in Wallace et al. [94] was developed. This index is evidence-based and serves as a measure of our confidence about EBSA likelihood and threats score assigned to individual seamounts. This index is calculated independently for EBSA likelihood and threats score.

Two measures were incorporated in the index: a data quality index (DQ) and a data deficiency index (DD). Data quality reflects origin and nature of the collected data and was divided into three categories: low (scored as 1), medium (scored as 0.5), and high (scored as 0) data quality. Considering the wide nature of seamount studies, the definition of these categories was not very strict but was based on general guidelines. The high data quality category was designed to include information mainly derived from rigorous scientific surveys. Even though data included in this category should be predominantly quantitative, qualitative data may also be considered as high quality data if in great detail. Medium quality data are incomplete quantitative information or qualitative descriptions of EBSA indicators and human impacts present on individual seamounts. These data should always be specific to a particular feature and validated in the literature. Low quality data include undisclosed data regarding wide geographic areas which do not specifically address seamounts, information inferred from models or from different seamount properties or data not properly referenced. The detailed scoring standards used to assign the data quality scores to all EBSA and threat indicators are described in the supplementary information (Table S2 and S3). Data deficiency (DD) was defined as the proportion of threats or EBSA indicators lacking data. Data deficiency could range from 0 (information available for all threats and indicators) to 1 (no information available). DQ and DD are combined into a data uncertainty index associated with each final EBSA likelihood and impact score. Data uncertainty is the sum of the average DQ score and the DD score. The data uncertainty index has a minimum value of 0 (all factors scored with high data quality) and a maximum value approaching 2 (few factors scored with low data quality).

The data uncertainty index is visualized as error bars in plots of the seamount EBSA likelihood scores versus human threats scores. Minimum and maximum values of each final seamount EBSA likelihood and human threats score are calculated by subtracting and adding the data uncertainty index. The error bars can therefore potentially range up to two units above and below the original score. In this manner, a seamount lacking data and/or with low data quality is shown as possibly belonging to different EBSA likelihood and threat categories, reflecting the uncertainty of the outcome.

### Seamount EBSA Portfolio

Protecting all seamounts is neither particularly rewarding nor practically feasible considering the high variation that exists in terms of their ecology, geophysics and potential human impacts and the large number of seamounts present in the world’s oceans. Therefore, approaches that systematically highlight conservation priority areas for seamount ecosystems can constitute a valuable tool for marine management purposes. We hereby propose an approach that combines the likelihood of a seamount constituting an EBSA and the level of human impact posed to a submarine feature to locate priority areas for seamount conservation at global, regional and local scales. This methodology allows the classification of individual seamounts into four main conservation categories, which can help in optimizing management efforts toward the protection of the most suitable areas. The portfolio categories are: Low EBSA likelihood-Low threats; Low EBSA likelihood-High threats; High EBSA likelihood-Low threats; High EBSA likelihood-High threats. EBSA likelihood and threats for individual seamounts can be easily summarized and graphically compared.

Additionally this approach is designed in a way that helps in visualizing what parts of the ecosystems (e.g., benthic, benthopelagic or pelagic) are contributing to the EBSA score or being threatened by human induced activities. This is of paramount importance in identifying seamounts that may be ecologically or biologically significant for both the benthic and pelagic components of the ecosystem and in complementing preexisting conservation strategies with the protection of underrepresented seamount components.

### Case Studies and Methodology Test

In order to test the framework developed here, we have randomly assigned the presence or absence of the ten indicators developed in the EBSA scoring procedure and of the nine types of threats considered in the threats scoring procedure to 1000 dummy seamounts (i.e. hypothetical seamounts having randomly assigned configurations of EBSA and threat indicators). In this way it was possible to assess the ability of our framework to assign real world seamounts to the different portfolio categories considered. In addition, a set of eight seamounts from different geographical areas were selected as case studies for applying the present framework, six located in the Atlantic Ocean (Sedlo, Condor, Anton Dohrn, Rosemary and Josephine seamounts) and two located in the Gulf of Alaska (Cobb and Bowie seamounts). Data for the evaluation process were obtained by reviewing the existing literature [95] and were presented in detail (Information S2).

## Results

### Testing the Methodology

**Figure 2:**
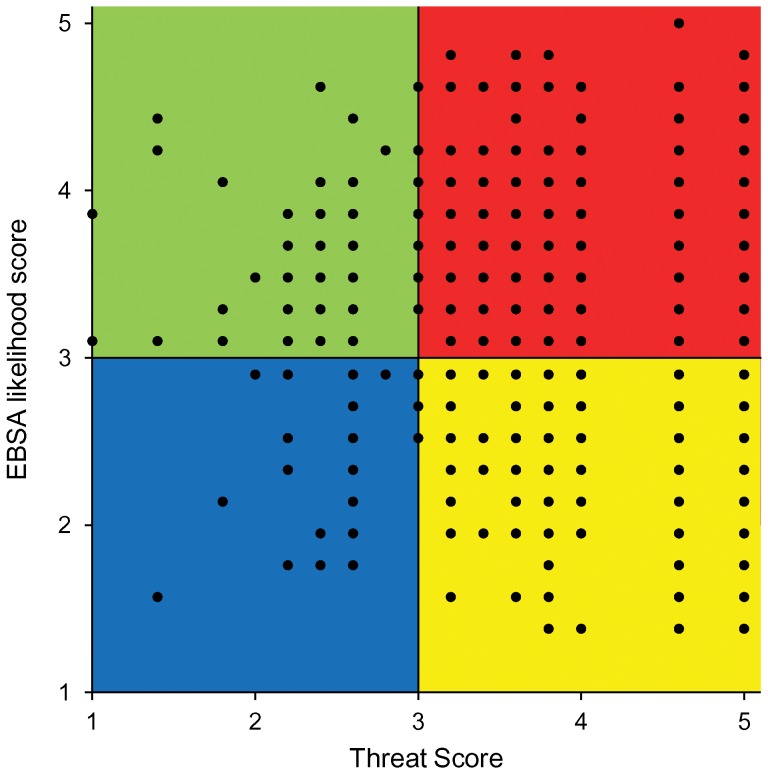
Seamount EBSA portfolio plot based on EBSA and threat scores randomly assigned to 1000 dummy seamounts. The different color represents four portfolio categories. Blue area: low EBSA likelihood-low threats exposure. Yellow area: low EBSA likelihood-high threats exposure. Green area: high EBSA likelihood-low threats exposure. Red area: high EBSA likelihood-high threats exposure.

The dummy seamounts were assigned to all 4 portfolio categories with only 5.1% seamounts considered as highly likely to be identified as EBSA with low degree of threat and 3.6% seamounts with low likelihood of being identified as EBSA with low degree of threat. Most of the dummy seamounts fall in the category low likelihood of being identified as EBSA with high degree of threat (36.2%) or high likelihood of being identified as EBSA with high degree of threat (55.1%). These results indicate that the framework is adequate to assign seamounts to different portfolio categories. In Figure 2 the outcomes of the framework can be visualized and seamounts compared allowing managers to prioritize their choices or policies in terms of protecting undisturbed areas, protecting disturbed areas for recovery of habitats and species, or both.

### Case Studies

For the 8 case study seamounts considered, seamount EBSA likelihood scores (Table 3) ranged from low for Rosemary seamount (2.5±0.59) to high for all other seamounts. Sedlo and Gorringe presented the highest EBSA likelihood scores (3.86±0.25 and 3.86±0.58, respectively). The uncertainty around these estimates is high for seamounts with low data quality and less attributes scored. For example, Josephine was identified as having a high likelihood of being a seamount EBSA but its score ranged from 2.59 to 3.99, i.e. from low to high likelihood of being an EBSA. Seamount threats scores (Table 4) ranged from low for Bowie (2.4±0.5), Sedlo (2.6±0.28) and Cobb (2.6±0.54), to high for Condor and Anton Dohrn seamounts (3.6±0.50 and 3.6±0.61, respectively) and were highest for Gorringe, Josephine and Rosemary seamounts (4.6±1.34, 5.0±1.16 and 5.0±0.61, respectively). Note the very high uncertainty around the threats estimates demonstrating the low data availability and quality.

**Table 3:** Seamount EBSA likelihood scores for the eight evaluated seamounts.

	Sedlo	Condor	Rosemary	Anton Dohrn	Josephine	Gorringe	Bowie	Cobb
EBSSA indicator	P/A (DQ)	P/A (DQ)	P/A (DQ)	P/A (DQ)	P/A (DQ)	P/A (DQ)	P/A (DQ)	P/A (DQ)
Vent communities	0 (L)	0 (M)	(DD)	(DD)	(DD)	(DD)	0 (M)	(DD)
Macrophytes	0 (H)	0 (M)	0 (H)	0 (H)	0 (L)	1 (H)	1 (M)	1 (M)
Cold-water coral reefs/gardens	1 (H)	1 (H)	1 (L)	1 (M)	1 (M)	1 (M)	0 (M)	0 (M)
Sponge aggregations	1 (H)	1 (H)	(DD)	1 (L)	1 (M)	1 (L)	0 (M)	0 (M)
Aggregating deep-sea fish	1 (H)	1 (M)	1 (H)	1 (H)	1 (H)	1 (M)	1 (M)	1 (M)
Threatened bottom fish or sharks	1 (H)	1 (M)	1 (H)	1 (H)	1 (M)	1 (M)	1 (M)	1 (M)
Threatened air-breathing visitors	1 (M)	− (−)	− (−)	1 (M)	1 (M)	1 (H)	1 (M)	1 (M)
Air-breathing visitors	− (−)	1 (M)	1 (M)	− (−)	− (−)	− (−)	− (−)	− (−)
Threatened visiting pelagics	1 (L)	1 (M)	(DD)	(DD)	(DD)	(DD)	1 (M)	1 (M)
Visiting pelagics	− (−)	− (−)	(DD)	(DD)	(DD)	(DD)	− (−)	− (−)
Naturalness	1 (H)	0 (M)	0 (M)	0 (L)	0 (M)	0 (M)	0 (M)	0 (H)
Shallow (above 800 m)	1 (H)	1 (H)	1 (H)	1 (H)	1 (M)	1 (H)	1 (H)	1 (H)
Seamount EBSA score	3.86	3.48	2.52	3.29	3.29	3.86	3.10	3.10
EBSA likelihood category	High	High	Low	High	High	High	High	High
Data uncertainty	0.25	0.35	0.59	0.58	0.70	0.58	0.45	0.49

The presence (1) or absence (0) of the seamount EBSA indicators is presented (P/A). Indicators with no information available were marked as data deficient (DD). Data quality (DQ) is showed as: H = high; M = medium; L = low. The seamount EBSA likelihood category and final data uncertainty score are also showed. Threatened-air-breathing/air-breathing and threatened-visiting-pelagics/visiting-pelagics are mutual exclusive and therefore only one will be scored while the other will be empty −(−).

**Table 4:** Threat scores for the evaluated seamounts.

	Sedlo	Condor	Rosemary	Anton Dohrn	Josephine	Gorringe	Bowie	Cobb
Human threat	P/A (DQ)	P/A (DQ)	P/A (DQ)	P/A (DQ)	P/A (DQ)	P/A (DQ)	P/A (DQ)	P/A (DQ)
Gillnet- bottom	0 (H)	0 (M)	1 (M)	0 (M)	1 (L)	(DD)	0 (M)	1 (M)
Hook and line	0 (M)	1 (M)	0 (M)	0 (M)	(DD)	(DD)	1 (M)	(DD)
Longline - bottom	0 (M)	1 (M)	1 (M)	1 (M)	1 (L)	1 (M)	1 (M)	1 (H)
Longline - pelagic	1 (L)	1 (M)	1 (M)	0 (M)	1 (L)	(DD)	0 (M)	(DD)
Pots and traps	0 (M)	0 (M)	1 (M)	1 (M)	(DD)	1 (L)	1 (M)	1 (H)
Purse seine	0 (H)	0 (M)	0 (M)	0 (M)	(DD)	1 (L)	0 (M)	(DD)
Trawl - bottom	0 (H)	0 (M)	1 (M)	1 (M)	1 (L)	1 (L)	0 (M)	0 (H)
Trawl - midwater	0 (H)	0 (M)	1 (M)	0 (M)	1 (L)	1 (L)	0 (M)	1 (H)
Mineral extraction	0 (H)	0 (M)	(DD)	(DD)	0 (H)	(DD)	0 (M)	(DD)
Mean threat score	2.60	3.60	5.00	3.60	5.00	4.60	2.40	2.60
Threat category	Low	High	High	High	High	High	Low	Low
Data uncertainty	0.28	0.50	0.61	0.61	1.16	1.34	0.50	0.54

The presence (1) or absence (0) of specific threats is showed (P/A). Threats with no information available were marked as data deficient (DD). Data quality (DQ) is showed as: H = high; M = medium; L = low. The threat category and final data uncertainty score are also showed.

**Figure 3:**
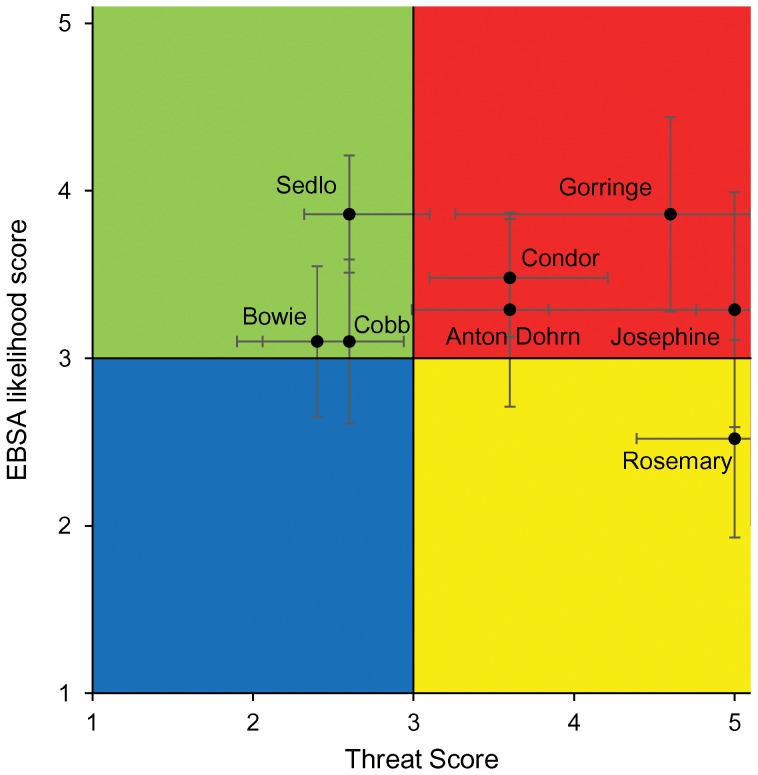
Seamount EBSA portfolio plot based on EBSA likelihood scores and threat scores for eight case studies. The different color represents four portfolio categories. Blue area: low EBSA likelihood-low threats exposure. Yellow area: low EBSA likelihood-high threats exposure. Green area: high EBSA likelihood-low threats exposure. Red area: high EBSA likelihood-high threats exposure. Error bars represent the data uncertainty index (see methods) proportional to data availability and quality.

**Figure 4:**
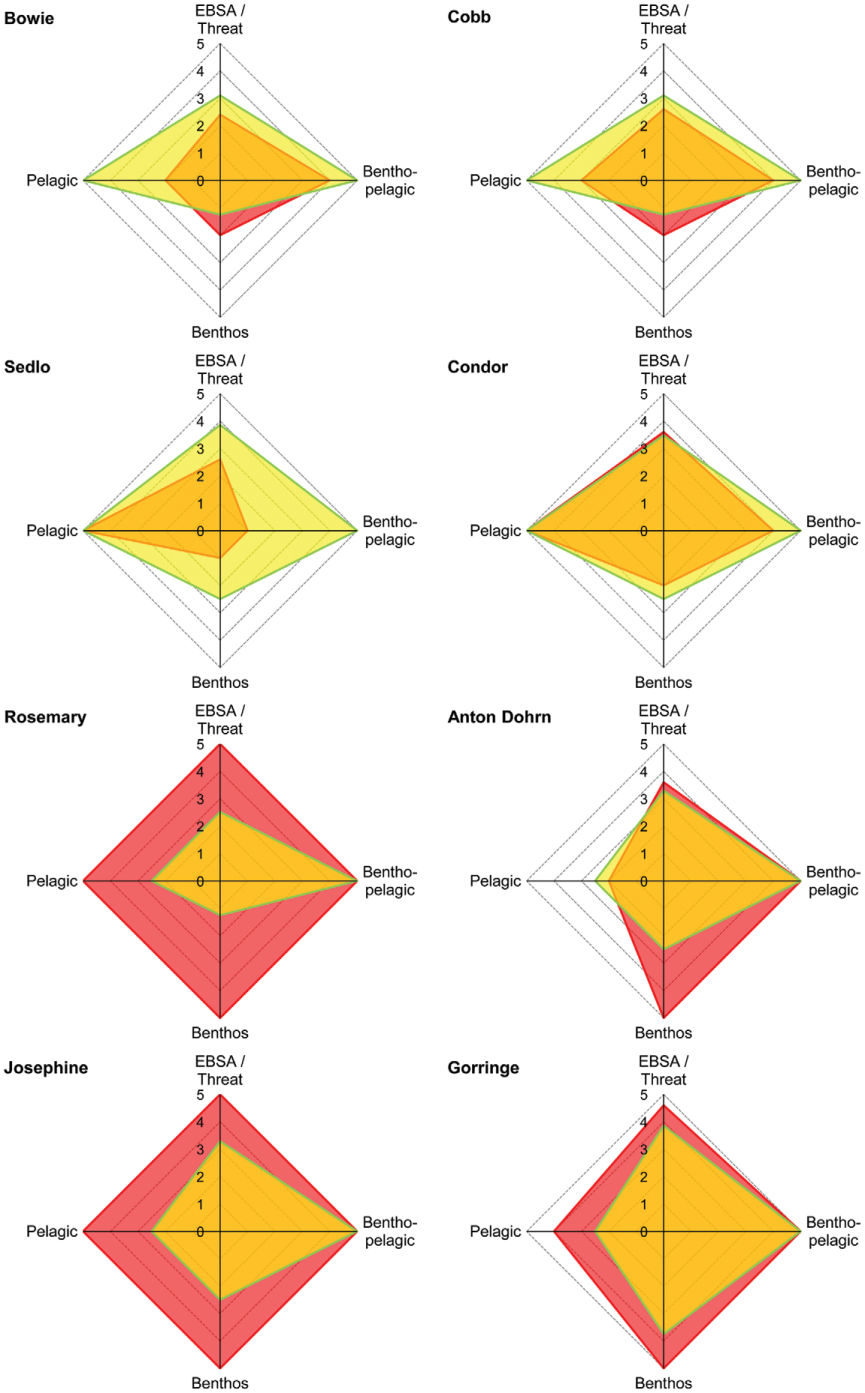
Components of the seamount ecosystem contributing to the EBSA score and its threat status. Radar plots for individual seamount showing what parts of the ecosystems (e.g. benthic, benthopelagic or pelagic) are contributing to the EBSA score (yellow area) or being threatened by human activities (red area). The EBSA component is shown as the proportion of attributes present (in a 0 to 5 scale) in each seamount as in Table 3, while the threats is given by the maximum threat estimated for each component of the seamount ecosystem. EBSA/Threat show the final EBSA and Threat score for each seamount.

Overall, the eight seamounts evaluated were allocated to three different portfolio categories of EBSA likelihood and threat exposure (Figure 3): high likelihood of being an EBSA-high threat exposure (Condor, Anton Dohrn, Gorringe and Josephine), high likelihood of being an EBSA-low threat exposure (Sedlo, Bowie and Cobb), and low likelihood of being an EBSA-high threat exposure (Rosemary). This framework also allow for the exploration of the parts of the ecosystem contributing for the definition of an EBSA or under major threat (Figure 4). For example, Gorringe seamount has a high likelihood of being an EBSA mainly because of its benthic and benthopelagic environments. The main threats posed to this seamount are also on deep-water corals and groundfish. On the other side, Sedlo seamount may be rich on benthopelagic communities but its groundfish or deep-water corals are not being impacted by human activities considered in this study. In fact, fishing on Sedlo seamount is exclusively based on longliners. The presence of pelagic longline fisheries on a seamount will results in a final threat score of 2.6 because of the high level of bycatch related to pelagic longlining [93]. However, considering the apparently low levels of bycatch in the Azorean fisheries e.g. [96], [97] this classification might overestimate the threats to Sedlo and other Azorean seamounts.

Of the case studies considered, Sedlo and Gorringe are those with the highest EBSA likelihood scores and, therefore, they may represent the most suitable areas where to adopt conservation measures. These two seamounts are experiencing very different levels of human pressure, with Sedlo presenting lower chances of detrimental effects caused by human activities than Gorringe. However, given the large uncertainty associated with the Gorringe threat score, a further evaluation of the activities should be undertaken. Sedlo has already been proposed as a suitable site for a marine protected area [98]. The preliminary outcomes of the present study seem to support this proposal highlighting a possible management strategy whose goals would be to protect a biologically and ecologically valuable area and, at the same time, to limit eventual conflicts between conservation and socio-economic interests. On the contrary, at the moment no conservation measure is scheduled for Gorringe seamount. In planning future actions on these two seamounts it should also be considered that Gorringe is the only one hosting benthic primary producers (macrophytes) among the Atlantic seamounts considered in this analysis, while Sedlo seamount seems to have a higher relevance for pelagic organisms and is the only submarine feature, among those considered, having high naturalness.

## Discussion

In order to achieve the conservation goals established under the Convention on Biological Diversity, scientists from different fields were asked to define and apply criteria which can highlight marine areas of particular interest [99]. In this context, identification and management of submarine mountains suitable for protection may represent an important step toward the systematic preservation of deep sea habitats and open ocean waters. In fact, even though many aspects of seamount ecosystems persist unknown, mounting evidence shows that they might play a key role in sustaining the pelagic and benthic production and biodiversity of deep seas and open oceans [18], [19], [23], [28], [30].

The framework proposed here was designed to set priorities in seamount conservation and to help developing spatially explicit seamount management policies. In order to avoid the location of protected areas in places that contribute little to preserve ecosystem structures and processes, the biological or ecological value of specific areas should always be considered as a primary criterion for the identification of conservation priorities [55], [100]. However, this criterion alone is not sufficient. The implementation of protected areas has to be included in a wider management context which integrates bio-ecological, economic and social goals to be successful [101]–[103]. The present framework, by considering both the conservation value of different seamounts with respect to the EBSA criteria and the importance of specific areas to human activities, represents one of the few attempts to implement an ecosystem approach to management in the deep sea. It allows, in fact, the definition of different strategies based on the governance objectives. For example, if there is the intention of restoring damaged ecosystems, seamounts having both high EBSA likelihood and high human threats scores will be chosen for conservation (uppermost right part of Figure 2). An alternative management policy similar to what was approved by CBD might focus toward the preservation of pristine areas with low levels of fishing and mining, where the likelihood of interactions between human activities and seamount EBSAs is low (uppermost left section of Figure 2) [9]. The case studies Sedlo and Gorringe represent an example of these two strategies. The final choice of the most appropriate strategy should always be site-specific and should depend upon socio-economic (e.g., economic importance; economic replaceability of the site; etc.) and ecological factors (e.g., likelihood of recovery after the cessation of the human activities; etc.) and on the specific goals managers have [104]. Furthermore, management-related criteria (i.e., criteria measuring how feasible is to effectively manage a site to achieve conservation goals) should also be considered in the final selection of the most suitable sites for conservation [41].

Three aspects were central in the practical definition of the proposed methodology. Our first concern was to develop a system which could provide solid measures of the relative value and threat status of individual seamounts. The choice of the selected seamount EBSA indicators and the definition of the most relevant human activities to seamount ecosystems were based on an extensive review of the existing literature and through intense consultations with seamount experts. This approach constitutes, therefore, a complete synthesis of what is presently known regarding seamount ecosystems. Our second concern was to design a system compatible with the data currently available. The major constraint faced by this kind of analysis is generally the scarcity of information readily available. In fact, while a very small portion of submarine relief has a fairly detailed ecological and biological description and an accurate report of ongoing anthropogenic activities, the large majority of seamounts have either never been explored or even charted through direct scientific measurements or only partially described [95]. The development of a methodology which could evaluate the relative importance and the threat status of individual features based on presence/absence data and deal with data deficiencies represented an attempt to overcome these limitations. The use of global models or questionnaires addressed to seamount experts may furnish information regarding the human activities currently present on individual features and allow the application of the present framework in area where little information is available, while a data quality index shows the confidence we have about any outcome provided. Finally, particular care was paid to keep the results simple to visualize and understand (Figures 2 and 3) in order to facilitate their implementation in future management actions.

The outcomes of the dummy seamounts may serve as an indication of the robustness of our methodology. Since our analysis was based only on presence/absence data, conservative outcomes regarding the threat status of individual seamounts should be expected. This is reflected in the outcomes of the analyses, where the highest proportion of seamounts fell within the high threat score category. Moreover, the consistent allocation of the dummy seamounts into high and low EBSA likelihood categories, as shown in Figure 2, is important to effectively highlight seamount areas of particular importance for conservation and to speed up management actions. Finally the combination of EBSA and threat scores identified seamounts belonging to all the four main portfolio categories indicating that the framework is adequate to assign seamounts to different portfolio categories.

Another important characteristic of this framework is that allows the identification of seamount EBSAs and threats considering different ecological groups in the pelagic and benthic realms (see example in Figure 4). This is a major step forward in the integration of these often segregated parts of the ecosystem and may allow managers to complement pre-existent conservation measures and to selectively mitigate the negative effects of human activities on particularly relevant seamount components. This framework will also allow the identification of seamounts with high data uncertainty and thus in urgent need of research. The methodology proposed here may constitute an important step forward in the implementation of conservation measures in deep see habitats and open ocean waters and help to fulfill the international commitments signed under the CBD. The simplicity of its scoring procedure and the nature of the data required to perform the analysis make it easy to understand and implement in actual conservation actions. Its systematic application at local scales and in different biogeographic provinces may enhance the conservation status of marine areas difficult to manage and ensure the protection of a wide range of habitats and organisms (both benthic and pelagic).

Future improvements to the methodology may consider the inclusion of additional threats such as climate change and pollution and tailor the threats score on a regional basis. Additionally, better quantitative assessment of the threats posed to a seamount could be implemented by including, for example, year when activities started, a measure of fishing effort and prospective mining areas. Currently, we assume that the presence of a particular threat would always have the same effects on the considered ecological groups which likely is a simplification. Future improvements to the framework should also take into account spatial and temporal patterns. In fact, both seamount EBSA indicators and human activities are not constant in time and space. However, detailed knowledge of seamount ecosystems and long time series data with high spatial and temporal resolution are required. At the moment, with a few exceptions, these conditions cannot be met.

Serious doubts regarding the sustainability of seamount trawl fishing and mining have been raised several times in the scientific community e.g. [49], [51], [53]. Increasing human pressure and poor knowledge of seamount ecosystems leave us little room to effectively direct conservation actions. Therefore, this framework which attempts to synthesize the best information currently available and guide conservation actions on the basis of ecological and economic values may represent an important tool to mitigate the biodiversity loss of one of the most representative deep water ecosystems. Its capability of highlighting seamount areas of particular importance, coupled with the spatial assessment of two key activities such as mining and fishing, may constitute the first step toward the implementation of representative and viable networks of marine protected areas [9].

## Supporting Information

Table S1 **List of habitat-forming Hexacorallia, Octocorallia and hydroids (adapted from Roberts** ***et al*** **. 2009).** In bold the most conspicuous cold-water taxa.
(DOCX)

Table S2 **Scoring standards used to assess the data quality of the EBSSA indicators.**
(DOCX)

Table S3 **Scoring standards used to assess the information quality regarding the human activities occurring at a seamount.**
(DOCX)

Information S1 **Guidelines for scoring human-induced threats to individual seamounts.**
(DOCX)

Information S2 **Description of the information used to evaluate the seamounts’ EBSA likelihood and threats.**
(DOCX)
