# Supplementary material for: An Ecosystem Evaluation Framework for Global Seamount Conservation and Management
Source: PLoS One. 2012 Aug 8;7(8):e42950. doi: 10.1371/journal.pone.0042950 (PMC3414466; doi:10.1371/journal.pone.0042950)
Supplement: Table S1 — List of habitat-forming Hexacorallia, Octocorallia and hydroids (adapted from Roberts et al . 2009). In bold the most conspicuous cold-water taxa. (DOCX) [file pone.0042950.s001.docx]

**Table S1. List of habitat-forming Hexacorallia, Octocorallia and hydroids (adapted from Roberts *et al*. 2009).** In bold the most conspicuous cold-water taxa.

| **Subclass** | **Order** | **Family** | **Genus** | **Species** |
| --- | --- | --- | --- | --- |
| Hexacorallia | Scleractinia | Pocilloporidae | *Madracis* | *myriaster* |
|  |  |  | *Madracis* | *interjecta* |
|  |  | Oculinidae | ***Oculina*** | ***varicosa*** |
|  |  |  | ***Madrepora*** | ***oculata*** |
|  |  |  | *Madrepora* | *carolina* |
|  |  | Caryophylliidae | ***Lophelia*** | ***pertusa*** |
|  |  |  | ***Solenosmilia*** | ***variabilis*** |
|  |  |  | *Desmophyllum* | *dianthus* |
|  |  |  | ***Goniocorella*** | ***dumosa*** |
|  |  |  | *Pourtalosmilia* | *conferta* |
|  |  |  | *Anomocora* | *fecunda* |
|  |  |  | *Coenosmilia* | *arbuscula* |
|  |  |  | *Cladocora* | *debilis* |
|  |  | Dendrophylliidae | ***Enallopsammia*** | ***profunda*** |
|  |  |  | *Enallopsammia* | *rostrata* |
|  |  |  | *Dendrophyllia* | *alternata* |
|  |  |  | *Dendrophyllia* | *minuscula* |
|  | Zoanthidea | Gerardiidae | *Gerardia* |  |
|  | Antipatharia | Antipathidae | *Antipathes* | *atlantica* |
|  |  |  | *Antipathes* | *caribbeana* |
|  |  |  | *Antipathes* | *curvata* |
|  |  |  | *Antipathes* | *dichotoma* |
|  |  |  | *Antipathes* | *dendrochristos* |
|  |  |  | *Antipathes* | *grandis* |
|  |  | Aphanipathidae | *Aphanipathes* |  |
|  |  | Cladopathidae | *Chrysopathes* |  |
|  |  |  | *Sibopathes* |  |
|  |  |  | *Trissopathes* |  |
|  |  | Stylopathidae | *Stylopathes* | *americana* |
|  |  |  | *Tylopathes* |  |
|  |  | Myriopathidae | *Plumapathes* | *pennacea* |
|  |  |  | *Tanacetopathes* | *hirta* |
|  |  | Schizopathidae | *Bathypathes* | *alternata* |
|  |  |  | *Dendropathypathes* | *boutillieri* |
|  |  |  | *Lillipathes* |  |
|  |  |  | *Parantipathes* | *tetrasticha* |
|  |  |  | *Stauropathes* |  |
|  |  | Leiopathidae | *Leiopathes* | *acanthphora* |
|  |  |  | *Leiopathes* | *glaberrima* |
| Octocorallia | Gorgonacea | Paragorgiidae | ***Paragorgia*** |  |
|  |  | Coralliidae | ***Corallium*** |  |
|  |  |  | *Paracorallium* |  |
|  |  | Plexauridae | *Alaskagorgia* | *aluetiana* |
|  |  |  | *Paramuricea* | *grandis* |
|  |  | Chrysogorgiidae | *Metallogorgia* |  |
|  |  |  | *Iridogorgia* |  |
|  |  | Primnoidae | ***Primnoa*** |  |
|  |  |  | *Callogorgia* |  |
|  |  |  | *Calyptrophora* |  |
|  |  |  | *Narella* |  |
|  |  |  | *Paracalyptrophora* |  |
|  |  |  | *Plumarella* |  |
|  |  |  | *Thouarella* |  |
|  |  | Isididae | *Acanella* |  |
|  |  |  | ***Isidella*** |  |
|  |  |  | *Keratoisis* |  |
| Hydroidolina | Anthoathecata | Stylasteridae | *Calyptrophora* | *reticulata* |
|  |  |  | *Distichopora* | *sulcata* |
|  |  |  | *Errina* | *antarctica* |
|  |  |  | *Errinopora* | *nanneca* |
|  |  |  | *Errinopsis* | *reticulum* |
|  |  |  | *Stenohelia* | *concinna* |
|  |  |  | *Stylaster* | *brochi* |
|  |  |  | *Stylaster* | *californicus* |
|  |  |  | *Stylaster* | *campylecus* |
|  |  |  | *Stylaster* | *cancellatus* |
|  |  |  | *Stylaster* | *densicaulis* |
|  |  |  | *Stylaster* | *eguchii* |
|  |  |  | *Stylaster* | *erubescens* |
|  |  |  | *Stylaster* | *ﬁlogranus* |
|  |  |  | *Stylaster* | *miniatus* |

**References**

Roberts JM, Wheeler AJ, Freiwald A, Cairns SD (2009) Cold-water corals: the biology and geology of deep-sea coral habitats. Cambridge: Cambridge University Press. 334 p.
