# Supplementary material for: An Ecosystem Evaluation Framework for Global Seamount Conservation and Management
Source: PLoS One. 2012 Aug 8;7(8):e42950. doi: 10.1371/journal.pone.0042950 (PMC3414466; doi:10.1371/journal.pone.0042950)
Supplement: Table S2 — Scoring standards used to assess the data quality of the EBSSA indicators. (DOCX) [file pone.0042950.s002.docx]

**Table S2. Scoring standards used to assess the data quality of the EBSSA indicators.**

| **EBSA criteria** | **EBSSA factor** | **Well known** | **Known** | **Inferred** |
| --- | --- | --- | --- | --- |
| **C1, C4, C5** | **Vent communities** | Detailed descriptions of the chemosynthetic community including qualitative data on the relation among chemosynthetic and non-chemosynthetic organisms | Descriptions of chemosynthetic organisms from the seamount, or qualitative description of the community reported by expert | Description of hydrothermal activity, but no description of any chemosynthetic organisms |
| **C1, C5, C6** | **Macrophyte meadows** | Macrophyte meadows are reported in several and/or detailed studies based on quantitative data | Data on macrophyte meadows are available from a limited number of studies or from a small proportion of the seamount, or reported by expert | The presence of macrophytes is inferred from models, anonymous entries in datasets, bycatch data from the proximity of the seamount or undisclosed data from several areas |
| **C3, C4, C6** | **Cold water coral**  **reefs/ gardens** | Cold water coral reefs/gardens are reported in several and/or detailed studies based on quantitative data | Data reporting cold water coral reefs/gardens are available from a limited number of studies or from a small proportion of the seamount, or reported by expert | The presence of coral aggregations is inferred from the presence of habitat-forming corals (S1), models, anonymous entries in datasets, bycatch data from the proximity of the seamount or undisclosed data from different areas |
| **C3, C4, C6** | **Sponge aggregations** | Sponge aggregations are reported in several and/or detailed studies based on quantitative data | Data reporting sponge aggregations are available from a limited number of studies or from a small portion of the seamount, or reported by expert | The presence of sponge aggregations is inferred from single specimens, models, anonymous entries in datasets, bycatch data from the proximity of the seamount or undisclosed data from several areas |
| **C2, C3** | **(Threatened) Air-breathing visitors*** | The seamount is shown to be important for air-breathing visitors for feeding, reproductive and/or migratory purposes in studies based on quantitative data or very detailed qualitative data | The presence of visiting air-breathing species on seamounts is reported in single or few studies, or reported by expert. The causes of the association are not clear | The presence of air-breathing visitors is inferred from anonymous entries in datasets, single observations, bycatch data from the proximity of the seamount or undisclosed data from several areas |
| **C2, C3** | **(Threatened) Large pelagic visitors*** | The seamount is shown to be important for large pelagic visitors for feeding, reproductive and/or migratory purposes in studies based on quantitative data or very detailed qualitative data | The presence of visiting large pelagic species on seamounts is reported in single or few studies, or reported by expert. The causes of the association are not clear | The presence of large pelagic visitors is inferred from anonymous entries in datasets, single observations, bycatch data from the proximity of the seamount, or undisclosed data from several areas |
| **C2, C4** | **Aggregating**  **deep sea ﬁsh** | The seamount is shown to be important for aggregating deep sea fish species in studies comparing seamount and non-seamount areas and/or in detailed studies based on quantitative data | Data on aggregating deep sea fish species are available from a limited number of studies or from expert knowledge | The presence of aggregating deep sea fish species is inferred from anonymous entries in datasets, bycatch data from the proximity of the seamount or undisclosed data from several areas |
| **C3** | **Threatened bottom fish and sharks** | The seamount is shown to be important for threatened deep-sea fish or shark species in studies comparing seamount and non-seamount areas and/or in detailed studies based on quantitative data | The presence of threatened deep-sea fish or shark species is reported in a limited number of studies, or reported by expert | The presence of threatened deep-sea fish or shark species is inferred from anonymous entries in datasets, bycatch data from the proximity of the seamount or undisclosed data from several areas |

*If species listed as near threatened (NT), vulnerable (VU), endangered (EN) or critically endangered (CR) in the IUCN Red List of Threatened Species (IUCN 2011) are present, the categories "threatened air-breathing visitors" and "threatened large pelagic visitors" are used, employing the same data quality standards.

**References**

IUCN (2011) IUCN Red List of Threatened Species. Version 2011.2. Available: http://www.iucnredlist.org. Accessed 27 April 2012.
