# Supplementary material for: An Ecosystem Evaluation Framework for Global Seamount Conservation and Management
Source: PLoS One. 2012 Aug 8;7(8):e42950. doi: 10.1371/journal.pone.0042950 (PMC3414466; doi:10.1371/journal.pone.0042950)
Supplement: Table S3 — Scoring standards used to assess the information quality regarding the human activities occurring at a seamount. (DOCX) [file pone.0042950.s003.docx]

**Table S3. Scoring standards used to assess the information quality regarding the human activities occurring at a seamount.**

| **Human impacts** | **Well Known** | **Known** | **Inferred** |
| --- | --- | --- | --- |
| **Fisheries** | Detailed description of fishing gears, catches and/or fishing effort on individual seamount (e.g. fishery reports, peer-reviewed publications), from the last 10 years | Description of the fishing gears acting on a seamount, but no quantitative (catch and/or effort) data provided (e.g.: descriptive literature, personal communications, grey literature) | Fishing gears, catches and /or effort extrapolated from report covering large areas (e.g. FAO areas) with no specific focus on the considered seamounts, or inferred from data older than 10 years |
| **Mining** | Detailed description of mining activities, including quantitative data, from the last 10 years | Description of mining activities on the seamount, but no quantitative data provided (e.g.: personal communication, grey literature) | Mining activities inferred from data covering large areas, with no specific focus on the considered seamounts |
