# Supplementary material for: An Ecosystem Evaluation Framework for Global Seamount Conservation and Management
Source: PLoS One. 2012 Aug 8;7(8):e42950. doi: 10.1371/journal.pone.0042950 (PMC3414466; doi:10.1371/journal.pone.0042950)
Supplement: Information S1 — Guidelines for scoring human-induced threats to individual seamounts. (DOCX) [file pone.0042950.s004.docx]

**Supporting Information S1. Guidelines for scoring human-induced threats to individual seamounts.**

The “threat score” for Bowie Seamount is calculated here as an example. According with the description reported in S4, only hook and line, pots and traps and bottom long-line fishing are present on this seamount. The set of activities are used to evaluate the impacts of each fishing gear on the benthic, benthopelagic and pelagic groups considered (see Figure 1 in the main text). The maximum impact potentially received by each ecological group will determine the threat status of the groups. In this case, bottom long-line constitute the highest threat for groundfish, cold water corals and sponges meadows potentially posing a high impact on the former and a low impact on the latter group. Bottom longlines, together with pots and traps, also constitute the highest threat for the physical habitat posing a low impact. Hook and line and, again, bottom long-line fishing are more likely to affect air-breathing visitors (low impact), while hook and line has the highest impact on large pelagic fish and sharks (low impact) (Table S2.1). In order to have the final “threat score” the maximum impact of the anthropogenic activities on the different ecological groups are averaged.

**Table. Impact of fishing gears and mining activities present on Bowie seamount on five ecological groups**. Null values indicate that a particular activity is absent. The maximum impacts on each ecological group are also shown and are averaged to calculate the final threat score.

| **Human threat** | **P/A (DQ)** |  | **Physical** | **CWC, sponges** | **Groundfish** | **Large pelagics** | **Air-breathing** |
| --- | --- | --- | --- | --- | --- | --- | --- |
| Gillnet- bottom | 0 (M) |  | 0 | 0 | 0 | 0 | 0 |
| Hook and line | 1 (M) |  | 1 | 1 | 3 | 2 | 2 |
| Longline - bottom | 1 (M) |  | 2 | 2 | 4 | 1 | 2 |
| Longline - pelagic | 0 (M) |  | 0 | 0 | 0 | 0 | 0 |
| Pots and traps | 1 (M) |  | 2 | 1 | 2 | 1 | 1 |
| Purse seine | 0 (M) |  | 0 | 0 | 0 | 0 | 0 |
| Trawl - bottom | 0 (M) |  | 0 | 0 | 0 | 0 | 0 |
| Trawl - midwater | 0 (M) |  | 0 | 0 | 0 | 0 | 0 |
| Mineral extraction | 0 (M) |  | 0 | 0 | 0 | 0 | 0 |
| Max impacts |  |  | 2 | 2 | 4 | 2 | 2 |
| Threat score | 2.4 |  |  |  |  |  |  |
