# Supplementary material for: An Ecosystem Evaluation Framework for Global Seamount Conservation and Management
Source: PLoS One. 2012 Aug 8;7(8):e42950. doi: 10.1371/journal.pone.0042950 (PMC3414466; doi:10.1371/journal.pone.0042950)
Supplement: Information S2 — Description of the information used to evaluate the seamounts’ EBSA likelihood and threats. (DOCX) [file pone.0042950.s005.docx]

**Supporting Information S2.** **Description of the information used to evaluate the seamounts’ EBSA likelihood and threats.**

**Sedlo seamount (40°25’N / 26°55’W)**

Sedlo is an isolated seamount in the Northern part of the Azorean section of the Portuguese EEZ, about 180 km northeast of the closest island, Graciosa. It is an elongated feature with three peaks which rises from a depth of 2500 to a minimum depth of 750 meters below sea surface [1]. Sedlo seamount has recently been part of the OASIS project [2]. This three-year research initiative assessed oceanographic, geochemical and biological aspects of Sedlo and Seine seamounts (the latter situated northeast of Madeira). Following the project, Sedlo was declared a marine protected area, integrated in the OSPAR network of MPAs [3].

Six seamount EBSA indicators were identified at Sedlo seamount (see Table 3 in the main text). Recent benthic surveys showed a high diversity of sessile megabenthos, which may form dense aggregations (mainly Hexacorallia – *Madrepora oculata –* and sponges) on the prevalently rocky surface of its summit [4]. Investigations of the demersal fish community recorded large reproductive aggregations of both alfonsino *(Beryx splendens*) and black cardinal fish (*Epigonus telescopus*) [5], two species recognized as typical seamount aggregating deep-sea fish [6,7]. These observations make Sedlo seamount the only known reproductive area for alfonsino and black cardinal fish in the Azores [3]. Several threatened deep-sea shark species were also recorded, such as the leafscale gulper shark (*Centrophorus squamosus*) and the Portuguese dogfish (*Centroscymnus coelolepis*) [5,8], considered as vulnerable and near threatened, respectively [9]. There is currently less established evidence regarding pelagic visitors to Sedlo seamount. Blue sharks (*Prionace glauca*) are fished in the surrounding area, and various air-breathing visitors, such as the endangered fin whale (*Balaenoptera physalus*) and sei whale (*Balaenoptera borealis*) [9] have been observed in the vicinity of the seamount [3]. However, no studies have assessed which role the seamount plays for these visitors. Since the minimum depth of the summit is at a 750 meters the presence of photosynthetic organisms such as macrophytes can be excluded. No indications of hydrothermal activity were found during the photographic surveys visiting the seamount [4], but since a part of the seamount remains unexplored, the presence of vent communities cannot be excluded with full confidence.

From a recent review [3] it appears that few human activities occur around Sedlo seamount. Bottom trawl and deep-water gillnet fisheries are currently banned from the entire Azorean section of the Portuguese EEZ, and the seamount’s isolated position and deep summit are likely to exclude hook and line, pots and traps and bottom longline fisheries. Pelagic longliners are present in the area, but no specific catch data are available [3]. The lack of known mineral resources on the seamount and its protection status are likely to prevent any mineral extraction in the near future.

**Condor seamount (38°33’N / 29°02’W)**

Condor is a ridge-shaped seamount reaching a minimum depth of about 200 meters. It is situated only 17 km Southwest of Faial Island (Azores). In 2009, local authorities, fishermen and scientist agreed upon a temporary non-fishing zone at the Condor seamount. This allowed for research projects employing long-term underwater observations to assess the functioning of both pelagic and benthic seamount components [10]. The seamount EBSA indicators observed at the Condor seamount include both coral gardens and sponge aggregations, with the densest coral gardens occurring on the shallower parts of the seamount [11]. No vent communities or macrophytes are known to be present on the seamount (Porteiro, personal communication). Fishery surveys have recorded the presence of aggregating deep-sea fish and threatened bottom and pelagic sharks (Menezes, pers. comm.). Even though no published data were found, air-breathing marine species are known to visit the seamount.

With the exception of the recent two-year fishery moratorium, Condor seamount was a common fishing ground, visited by local longline and hook and line fisheries [11] (Porteiro personal communication). As for other seamounts in the Azorean section of the Portuguese EEZ, no bottom trawl or net fisheries are allowed, and no mining activities are currently occurring in the area.

**Anton Dohrn (57°28’N / 11°05’W) and Rosemary Bank (59°12’N / 10°15’W) seamounts**

Three large seamounts are found in the Rockall Trough, the deep-water basin between the British shelf slope and the Rockall plateau: Hebrides Terrace, Anton Dohrn and Rosemary Bank [12]. These features rise from a basin depth of around 2200 meters to summit depths ranging from 1000 meters (the Hebrides Terrace seamount) to around 500 meters (Anton Dohrn and Rosemary Bank seamounts). Anton Dohrn seamount has a gently domed summit with 100 meter thick sediment cover, while the summit of Rosemary Bank is scattered by pinnacles reaching a minimum depth of 350 meter. Both seamounts have been the focus of various investigations, including recent biological surveys [13-15]. On the contrary, no available information regarding the biology of the Hebrides Terrace Seamount was found. The Hebrides Terrace Seamount was therefore not considered in the present evaluation. Cold-water coral reefs have been observed on the flanks of Anton Dohrn seamount, in addition to abundant gorgonians, antipatharians and sponges [14]. Whether these sponges form aggregations was not specified. The description of the benthic organisms present on the Rosemary Bank was limited to records of unknown species Scleractinia [16]. Fishery surveys conducted on Anton Dohrn and Rosemary Bank seamounts registered aggregating deep-sea fish and threatened deep-sea sharks (*Chimaera monstrosa*) in both areas [13]. Regarding air-breathing visitors, various seabirds and marine mammals have been sighted around these two seamounts, including the endangered fin whale (*Balaenoptera physalus*) at Anton Dohrn seamount [17].

Deepwater trawl fisheries have been operating in The Rockall Trough area since the 1970s, first by German and later by French vessels [18]. Several fish stocks are considered overexploited, suggesting that the present fishing pressure is unsustainable [18]. Still, a range of different EU and UK fisheries currently operate on the seamounts in the Rockall Trough employing both demersal and pelagic gears. Presence/absence data of the fishing gears operating on the evaluated seamounts were only available from 2006 to 2009, but specific catch data could not be obtained. No information was found regarding mineral deposits on these features.

**Josephine seamount (36°40’N / 14°15’W)**

Josephine seamount is the tallest feature of the Madeira-Tore Rise, a submarine ridge in the central east Atlantic [19]. Discovered in 1869, it might have been the first seamount recognized as a direct result of oceanic explorations [20]. Although Josephine seamount is situated beyond areas of national jurisdiction it has been subject to numerous geological and biological investigations. It has currently no official legal status but has been recently identified as a candidate EBSA for the North-East Atlantic principally due to its high productivity and vulnerability [21]. In 2010 the Ministerial Meeting of the OSPAR Commission adopted the OSPAR Decision 2010/5 to establish a High Sea MPA in the water column above Josephine [22]. In addition, the Portuguese government has proposed a protected area on the seamount itself, in conjunction with a submission presented to the Commission on the Limits of the Continental Shelf to include the seabed of this area in the Portuguese territory. Various species of solitary scleractinians and gorgonians were recorded from this seamount [22,23]. Some gorgonian and sponge species were reported to form dense aggregations [24]. However, no recent references confirmed the presence of coral gardens or sponge aggregations. Considering that the depth of the peak is at about 130 meters below surface [19], the presence of macrophytes is unlikely. The seamount’s fish community has been investigated by visual observations [25], which confirmed the presence of two aggregating deep-sea species: silver roughy (*Hoplostethus mediterraneus*, Trachichthyidae) and common mora (*Mora moro,* Moridae). The endangered bottlenose skate (*Rostroraja alba*, Rajidae) had previous been registered on the seamount. In addition, a recent acoustical study recorded a variety of marine mammals in the vicinity of the seamount, including vulnerable sperm whales (*Physeter macrocephalus*, Physeteridae) [26].

A Russian fishery for horse mackerel (*Trachurus trachurus,* Carangidae), mackerel (*Scomber* sp., Scombridae), and scabbardfish (family Trichiuridae) has operated on seamounts in the Madeira/Canary area, including Josephine seamount, since the early seventies, [27]. The main gear employed was midwater trawl, but bottom trawls and purse seines were also used in some seamount of the region. In addition, Portuguese longliners used to operate in the area [27]. No specific data regarding the fisheries currently operating on the Josephine seamount could be found. However, several fisheries are operating in this ICES subarea (IX), including bottom gillnet, bottom trawl and bottom longline [28]. No mineral exploitation is currently operating at Josephine seamount, and no valuable mineral resources have been discovered to date [22].

**Gorringe seamount (36°38’N / 11°18’W)**

The Gorringe Ridge is a large submarine bank situated at a basin depth of 5000 meters on the margin between the Eurasian and African plates, Southwest of Portugal, and within the Portuguese EEZ. This underwater ridge is composed of two seamounts (Ormonde and Gettysburg), both reaching the photic layer at depths of around 30-40 meters [29]. The non-governmental organization OCEANA recently summarized the biological information available from these seamounts [30]. Gorringe’s summit area is dominated by kelp and red gorgonians, with the additional presence of various reef-building (scleractinian) coral and sponge species [30,31]. The IUCN “near threatened”-listed giant manta (*Manta birostris*) have been recorded at the seamount and loggerhead sea turtles (*Caretta caretta*) are known to visit the zone [30,31].

The seamount is used as a fishing ground for the black scabbardfish (*Aphanopus carbo),* an aggregating deep sea fish species. Apart from this bottom longline fishery, little information is available about current human impacts. Indications of demersal fishing activities with pots were observed during recent research expeditions visiting the seamount [30]. Similarly as for other fishable seamounts in the region, Gorringe Ridge seamount has been the subject of Russian fisheries during the last decades of the 20^th^ century, employing mid- and bottom trawling and purse seine techniques. Therefore, the seamount is not in a pristine state.

**Bowie Seamount (53°19’N / 135°39’W)**

Bowie Seamount (also known as Sgaan Kinghlas in the Haida language), is an elongated relief that lies 180 km west of the Queen Charlotte Islands, Canada. It represents the youngest feature (≤ 0.7 Ma) of the Kodiak-Bowie Seamount Chain and one of the shallowest seamounts in Canada’s Pacific waters, having a small flat top at a depth of approximately 235 m studded with peaks rising up to 34 m below the sea surface [32]. Several authors reported the Bowie Seamount communities as biologically rich, however there is only little information characterizing its benthic and pelagic habitats [33]. The rocky substrate presents an extensive algal coverage, while corals and sponges have only been sporadically encountered and do not seem to form gardens or meadows. Data coming from catch logs and biological samples permit a preliminary description of the seamount fish assemblage. The rockfish dominate the fish community at Bowie Seamount (21 species) and include four aggregating deep-sea fishes: Bocaccio rockfish (*Sebastes paucispinis*, Sebastidae), Rosethorn rockfish (*Sebastes helvomaculatus*, Sebastidae), Yelloweye rockfish (*Sebastes ruberrimus*, Sebastidae) and Widow rockfish (*Sebastes entomelas*, Sebastidae) [33]. Other commercially important fishes reported are Pacific halibut (*Hippoglossus stenolepis*, Pleuronectidae) and Sablefish (*Anoplopoma fimbria*, Anoplopomatidae) and seasonally, albacore tuna (*Thunnus alalunga*, Scombridae). One benthopelagic and two pelagic species of sharks described as vulnerable or nearly threatened in the IUCN red list were found on the seamount: Piked dogfish (*Squalus acanthias*, Squalidae), Basking shark (*Cetorhinus maximus*, Cetorhinidae) and Blue shark (*Prionace glauca*, Carcharhinidae) [33]. Finally, several species of marine mammals (7 species) and seabirds (16 species), including six species listed as endangered, vulnerable or nearly threatened in the IUCN red list make the seamount particularly interesting for conservation [33]. Fishing activities have occurred around Bowie Seamount since the first half of the last century, the most relevant being associated with sablefish and rockfish, and in the past, halibut [33]. The establishment of an MPA around part of the Bowie seamount has banned most of these activities, even though bottom longline, pots and traps and hook and line fisheries are still allowed (<http://laws-lois.justice.gc.ca/eng/regulations/SOR-2008-124/index.html>). Mineral resources are so scarce in the area that mining does not constitute a threat for this seamount [33].

**Cobb Seamount (46°44’N / 130°48’W)**

Cobb Seamount is a flat-topped seamount located in the Gulf of Alaska, outside the U.S. Exclusive Economic Zone, about 500 km west of Gray’s Harbour, Washington [34]. Its summit plateau is between 200 and 300 m deep, but several peaks reach a minimum depth of 34 m [32]. As for the Bowie Seamount, patches of algae are found on its rocky substrate while no habitat forming corals or sponges are described on the seamount [35]. The Taylor cap that seems to surround this feature [36,37] may affect its primary production. However, no consistent evidences of increased primary production are available in literature [34,36,38,39]. Rockfish species are particularly abundant on this feature and, as for the Bowie Seamount, the aggregating deep sea fish species Bocaccio, Rosethorn, Yelloweye and Widow rockfish are present [34,35,40]. The benthic Big skate (*Raja binoculata*, Rajidae) and the pelagic Blue shark, listed as nearly threatened in the IUCN red list, were encountered in association with Cobb seamount [34,35]. The Albacore tuna (*Thunnus alalunga*, Scombridae) was occasionally reported in the proximity of the seamount [34]. Five species of seabirds were recorded in association with Cobb Seamount; in particular Black-footed albatross (*Phoebastria nigripes*, Diomedeidae) and Sooty shearwater (*Puffinus griseus*, Procellariidae) are two species listed as vulnerable and nearly threatened in the IUCN red list [41]. Fishing occurred on Cobb Seamount at least since the late 70s and mainly targeted widow rockfish and sablefish [42]. Bottom gillnets, bottom longlines, midwater trawls and pots and traps were the fishing gears most commonly employed, while the rough terrain does not allow the use of bottom trawls [42]. No information regarding mineral deposits is available for Cobb Seamount.

**References**

1. Christiansen B, Bashmachnikov I, José F (2005) The Bathymetry of Sedlo and Seine Seamounts. Internal OASIS report. Available: [http://www1.uni-hamburg.de/OASIS//Pages/publications/Bathymetry-Report.pdf](http://www1.uni-hamburg.de/OASIS/Pages/publications/Bathymetry-Report.pdf). Accessed: 28 February 2012.

2. Christiansen B, Wolff G (2009) The oceanography, biogeochemistry and ecology of two NE Atlantic seamounts: The OASIS project. Deep Sea Res II 56: 2579–2581.

3. Santos RS, Christiansen S, Christiansen B, Gubbay S (2009) Toward the conservation and management of Sedlo Seamount: A case study. Deep Sea Res II 56: 2720–2730.

4. OASIS (2006) Final Scientific and Technical Report for the period 01.12.2002-30.11.2005. Oceanic Seamounts: an Integrated Study, contract number EVK3-CT-2002-00073-OASIS. 52 p.

5. Menezes GM, Rosa A, Melo O, Pinho MR (2009) Demersal fish assemblages off the Seine and Sedlo seamounts (northeast Atlantic). Deep Sea Res II 56: 2683–2704.

6. Koslow JA (1996) Energetic and life-history patterns of deep-sea benthic, benthopelagic and seamount associated fish. J Fish Biol 49 (Suppl A): 54–74

7. Morato T, Cheung WWL, Pitcher TJ (2006) Vulnerability of seamount fish to fishing: fuzzy analysis of life history attributes. J Fish Biol 68: 209–221.

8. Melo O, Menezes G (2002) Exploratory fishing of the orange roughy (*Hoplostethus atlanticus*) in some seamounts of the Azores archipelago. ICES CM 2002/M:26.

9. IUCN (2011) IUCN Red List of Threatened Species. Version 2011.2. Available: http://www.iucnredlist.org. Accessed 5 December 2011.

10. Morato T, Pitcher TJ, Clark MR, Menezes G, Tempera F, et al. (2010) Can we protect seamounts for research? A call for conservation. Oceanography 23: 190–199.

11. Tempera F, Giacomello E, Mitchell NC, Campos AS, Henriques AB, et al. (2012) Mapping Condor Seamount Seafloor Environment and Associated Biological Assemblages (Azores, NE Atlantic). In: Harris PT, Baker EK, editors. Seafloor geomorphology as benthic habitat: Geohab Atlas of Seafloor Geomorphic Features and Benthic Habitats. London, UK: Elsevier. pp. 245-251.

12. Jacobs CL (2006) An appraisal of the surface geology and sedimentary processes within SEA7, the UK continental shelf. Southampton: National Oceanography Centre Research and Consultancy Report, No. 18. 127 p.

13. Neat F, Burns F, Drewery J (2008) The deepwater ecosystem of the continental shelf slope and seamounts of the Rockall trough: a report on the ecology and biodiversity based on FRS scientific surveys. Aberdeen, UK: Fisheries Research Services Internal Report No 02/08. 30 pp. Available: <http://www.scotland.gov.uk/Uploads/Documents/IR0208.pdf>. Accessed 28 February 2012.

14. Stewart H, Davies J, Long D, Strömberg H, Hitchen K (2009) JNCC Offshore Natura Survey: Anton Dohrn Seamount and East Rockall Bank.  2009/03-JNCC Cruise Report. Report No. CR/09/113. Available: <http://jncc.defra.gov.uk/PDF/2009_3_JNCC_Cruise_Report_Public%20%282%29.pdf>. Accessed 27 February 2012.

15. Howell KL, Mowles SL, Foggo A (2010) Mounting evidence: near-slope seamounts are faunally indistinct from an adjacent bank. Mar Ecol 31: suppl 152–162.

16. Zibrowius H (1980) Les Scléractiniaires de la Méditerranée et de l'Atlantique nord-oriental. Memoires de l'Institut Oceanographique Foundation Albert Ier, Prince de Monaco 11: 247 p.

17. Pollock CM, Mavor R, Weir CR, Reid A, White RW, et al. (2000) The distribution of seabirds and marine mammals in the Atlantic Frontier, north and west of Scotland. Aberdeen: JNCC. 92 p.

18. Gordon JDM (2003) The Rockall Trough, Northeast Atlantic: the cradle of deep-sea biological oceanography that is now being subjected to unsustainable fishing activity. Journal of Northwest Atlantic Fisheries Science 31: 57–83.

19. Geldmacher J, Hoernle K, Klugel A, van den Bogaard P, Wombacher F, et al. (2006) Origin and geochemical evolution of the Madeira-Tore Rise (eastern North Atlantic). J. Geophys. Res., 111: (B9)

20. Brewin PE, Stocks KI, Menezes G (2007) A history of seamount research. In: Pitcher TJ, Morato T, Hart PJB, Clark MR, Haggan N, et al., editors. Seamounts: ecology, fisheries and conservation. Blackwell Fisheries and Aquatic Resources Series 12. Oxford, UK: Blackwell Publishing. pp. 41- 61.

21. CBD (2012) Josephine Seamount. Available: <http://ebsa.cbd.int/ebsa-submissions/josephine-seamount>. Accessed 17 February 2012.

22. OSPAR (2011) Background Document on the Josephine Seamount Marine Protected Area. Report prepared by the OSPAR Intersessional Correspondence Group on Marine Protected Areas. Biological Diversity and Ecosystems, 551, 27.

23. WWF (2001) Implementation of the EU Habitats Directive Offshore: Natura 2000 sites for reefs and submerged sandbanks. Vol. IV. Godalming, Surrey: WWF-UK. 288

24. Rad U von (1974) Great Meteor and Josephine Seamounts (eastern North Atlantic): composition and origin of bioclastic sands, carbonate and pyroclastic rocks. Meteor Forschungsergebnisse C19: 1-61.

25. Pakhorukov NP (2008) Visual observations of fish from seamounts of the Southern Azores Region (the Atlantic Ocean). Journal of Ichthyology 48: 114–123.

26. Giorli G, Au WWL, Morrissey RP (2011) Passive acoustic monitoring of cetacean at Josephine Seamount, Portugal. J. Acoust. Soc. Am. Program abstracts, contributing paper 5aAB5 129: 2638-2638. Available: <http://asadl.org/jasa/resource/1/jasman/v129/i4/p2634_s1>. Accessed 10 February 2012.

27. Clark MR, Vinnichenko VI, Gordon JDM, Beck-Bulat GZ, Kukharev NN, et al. (2007) Large-scale distant-water trawl fisheries on seamounts. In: Pitcher TJ, Morato T, Hart PJB, Clark MR, Haggan N, et al., editors. Seamounts: ecology, fisheries and conservation. Blackwell Fisheries and Aquatic Resources Series 12. Oxford, UK: Blackwell Publishing. pp. 361-399.

28. ICES (2006) Report of the working group on the biology and assessment of deep-sea fisheries resources (WGDEEP). 2 - 11 May 2006, Vigo, Spain. 504 p. Available: [http://wwz.ifremer.fr/deepfishman/ content/download/17020/251155/file/wgdeep2006.pdf](http://wwz.ifremer.fr/deepfishman/%20content/download/17020/251155/file/wgdeep2006.pdf). Accessed 28 February 2012.

29. Alteriis GD, Passaro S, Tonielli R (2005) New, high resolution swath bathymetry of Gettysburg and Ormonde Seamounts (Gorringe Bank, eastern Atlantic) and first geological results. Marine Geophysical Researches 24: 223-244.

30. OCEANA (2005) The seamounts of the Gorringe Bank. 39 p. Available: [http://eu.oceana.org/sites /default/files/reports/seamounts_gorringe_bank_eng.pdf](http://eu.oceana.org/sites%20/default/files/reports/seamounts_gorringe_bank_eng.pdf). Accessed 28 February 2012.

31. Gonçalves JMS, Bispo J, Silva JA (2004) Underwater survey of ichthyofauna of eastern Atlantic seamounts: Gettysburg and Ormond (Gorringe Bank). Arch Fish Mar Res 51: 233–240.

32. Chaytor JD, Keller RA, Duncan RA, Dziak RP (2007) Seamount morphology in the Bowie and Cobb hot spot trails, Gulf of Alaska. Geochem Geophys Geosyst 8: 1-26.

33. Canessa RR, Conley KW, Smiley BD (2003) Bowie Seamount pilot marine protected area: an ecosystem overview. Can Tech Rep Fish Aquat Sci 2461: 85 p.

34. Budinger TF, Enbysk BJ (1960) Cobb Seamount, a deep-sea feature off the Washington coast. Seattle: Technical Report No. 60, Department of Oceanography, University of Washington. 88 p.

35. Parker T, Tunnicliffe V (1994) Dispersal Strategies of the biota on an oceanic seamount: implications for ecology and biogeography. Biol Bull 187: 336–345.

36. Dower JF, Freeland H, Juniper K (1992) A strong biological response to oceanic flow past Cobb Seamount. Deep-Sea Res Pt I 39: 1139–1145.

37. Freeland H (1994) Ocean circulation at and near Cobb Seamount. Deep Sea Res I. 41: 1715–1732.

38. Sime-Ngando T, Juniper K, Vézina AF (1992) Ciliated protozoan communities over Cobb Seamount: increase in biomass and spatial patchiness. Mar Ecol Prog Ser 89: 37-51.

39. Comeau LA, Vezina AF, Bourgeois M, Juniper K (1995) Relationship between phytoplankton production and the physical structure of the water column near Cobb Seamount, northeast Pacific. Deep Sea Res I 42: 993–1005.

40. Pearson DE, Douglas DA, Barss B (1993) Biological observations from the Cobb Seamount rockfish fishery. Fishery Bullettin 91: 573-576.

41. Thompson DR (2007) Air-breathing visitors to seamounts: Importance of seamounts to seabirds. In: Pitcher TJ, Morato T, Hart PJB, Clark MR, Haggan N, et al., editors. Seamounts: ecology, fisheries and conservation. Blackwell Fisheries and Aquatic Resources Series 12. Oxford, UK: Blackwell Publishing. pp. 245-251.

42. Douglas DA (2011) The Oregon shore-based Cobb Seamount Fishery, 1991-2003: catch summaries and biological observations. Astoria, Oregon: Oregon Department of Fish and Wildlife, Marine resource program. 28 p.
